# Characteristics of community savings groups in rural Eastern Uganda: opportunities for improving access to maternal health services

**DOI:** 10.1080/16549716.2017.1347363

**Published:** 2017-08-31

**Authors:** Aloysius Mutebi, Rornald Muhumuza Kananura, Elizabeth Ekirapa-Kiracho, John Bua, Suzanne Namusoke Kiwanuka, Gertrude Nammazi, Ligia Paina, Moses Tetui

**Affiliations:** ^a^ Makerere University School of Public Health, Department of Health Policy Planning and Management, Kampala, Uganda; ^b^ Makerere University Centre of Excellence for Maternal and Newborn Health Research, Kampala, Uganda; ^c^ Department of International Health, Johns Hopkins Bloomberg School of Public Health, Johns Hopkins University, Baltimore, MD, USA; ^d^ Epidemiology and Global Health Unit, Department of Public Health and Clinical Medicine, Umeå University, Umeå, Sweden

**Keywords:** MANIFEST - Maternal and Neonatal Implementation for Equitable Systems Study, Community-based savings groups, composition and management, implementation science, health insurance

## Abstract

**Background**: Rural populations in Uganda have limited access to formal financial Institutions, but a growing majority belong to saving groups. These saving groups could have the potential to improve household income and access to health services.

**Objective:** To understand organizational characteristics, benefits and challenges, of savings groups in rural Uganda.

**Methods**: This was a cross-sectional descriptive study that employed both quantitative and qualitative data collection techniques. Data on the characteristics of community-based savings groups (CBSGs) were collected from 247 CBSG leaders in the districts of Kamuli, Kibukuand Pallisa using self-administered open-ended questionnaires. To triangulate the findings, we conducted in-depth interviews with seven CBSG leaders. Descriptive quantitative and content analysis for qualitative data was undertaken respectively.

**Results**: Almost a quarter of the savings groups had 5–14 members and slightly more than half of the saving groups had 15–30 members. Ninety-three percent of the CBSGs indicated electing their management committees democratically to select the group leaders and held meetings at least once a week. Eighty-nine percent of the CBSGs had used metallic boxes to keep their money, while 10% of the CBSGs kept their money using mobile money and banks,respectively. The main reasons for the formation of CBSGs were to increase household income, developing the community and saving for emergencies. The most common challenges associated with CBSG management included high illiteracy (35%) among the leaders,irregular attendance of meetings (22%), and lack of training on management and leadership(19%). The qualitative findings agreed with the quantitative findings and served to triangulate the main results.

**Conclusions**: Saving groups in Uganda have the basic required structures; however, challenges exist in relation to training and management of the groups and their assets. The government and development partners should work together to provide technical support to the groups.

## Background

Although Uganda is said to have achieved the Millennium Development Goal one (MDG1) of halving poverty by reducing the national poverty headcount from 38.8% to 19.7% [1], the majority of rural households are still below the poverty levels [2]. Most households in rural areas depend on subsistence farming for their livelihood, i.e. for income generation and household welfare. Women, who largely have a comparative disadvantage when it comes to decision-making and resource ownership, suffer the most from limited access to finances, which greatly influences health care services utilization [3]. Access to cash affects women’s ability to afford transport costs and to meet the cost of personal needs, such as appropriate clothing while visiting health facilities, as well as the purchase of other items that are often required during pregnancy and childbirth [3–5].

In Uganda, just as in most low-income countries, financial services are largely inaccessible to rural populations [6–8] who are considered to have small transaction sizes and poor infrastructure that make it difficult for formal financial services to reach them [7,9]. Work by Finscope showed that 62% of people were excluded from financial services [8]. Efforts to increase access and availability of finance has largely been spearheaded by non-governmental organizations (NGOs) with minimal government involvement [10]. The available evidence indicates that such initiatives have contributed to the growth of informal saving groups [11] that are able to meet the needs of poorer segments of the population by providing them with savings-based rather than credit-based services at affordable interest rates [7]. Saving groups have been described as time-bound accumulating savings and credit associations, which distribute their assets, which are often financial in nature, to their members during a cycle, which runs for a specific period, and then it starts again [7]. It usually comprises a pool of people who reside in a similar geographical region [12]. The groups act as social safety nets for communities by providing quick financial credit at low interest rates [7,12]. They are also cheaper per person compared to other financial services [13]. Saving groups have also been noted to be able to facilitate the mobilization of large groups of people through the regular meetings and financial transactions that are conducted [12–14]. Therefore, they have been used to promote other development activities, such as health or literacy training, promotion of products, such as agricultural inputs and other social products [12–14]. These saving groups were initiated in the early 1990s and have now spread to a growing number of countries in south Asia (India, Bangladesh) and Africa (Burundi, Rwanda Uganda, Kenya) [7,12–14]. The factors that have enhanced the success of saving groups include easy access to the groups, good security, flexible savings, transparency and autonomy [7].

The organization and management of saving groups mainly hinges on their membership interests and reasons for formation. Most of the credit-based saving group (CBSGs) pool resources on a regular interval, loan out the pooled resources and share dividends at specified times. Groups are organized through a common understanding of the members, who are usually no more than 30 individuals [15]. Members usually elect a committee that is responsible for the daily administrative issues of running the group. Committee members are volunteers who are trusted by the members and usually serve for a specific period of time [16]. The money from saving groups is, typically, kept in a cash box that has three locks with keys kept by different members. This method has been generally safe, especially when the sums of money kept are small [7]. When the money increases the risk is minimized by distributing it among different members or keeping it in the bank [7].

Saving groups may be initiated by facilitating agencies or they may grow spontaneously [12–14]. When initiated by a facilitating agency, the groups are mentored and taught to offer financial services over a period of 8–12 months [13]. They are considered independent when they are able to run organized meetings, to manage their financial services (collection of savings, lending and sharing out of their dividends) and to have accurate records [7].

There is some evidence that saving groups have been used to promote access to health services [14]. However, the opportunities to enhance access to health services within savings groups has neither been explored comprehensively nor documented extensively [14,17].

It is, therefore, important to understand the structure and management of saving groups in rural communities and their potential in improving birth preparedness. This is especially important because many of the saving groups in rural communities of Uganda, like other low- and middle-income countries, have developed spontaneously and have shown a promise to improving the lives of the communities. This paper, therefore, seeks to describe the key characteristics of saving groups, benefits and challenges of CBSGs, as well as solutions to the challenges. It has also been written as a background to another paper in this supplement that focuses on how the MANIFEST programme explored the potential of saving groups to increase access to maternal health services [18].

## Methods

### Study area and design

This study was conducted in the districts of Kamuli, Pallisa and Kibuku in Eastern Uganda in 2013. At least 60% of the population in the Eastern region are illiterate and the majority (95%) are subsistence farmers [19]. This was a descriptive cross-sectional study that employed both qualitative and quantitative techniques using a semi-structured questionnaire. Data used in this article were part of the baseline information that was collected to inform the design of demand–creation strategies for the MANIFEST project, which was implemented between 2013 and 2015.

### The savings group under MANIFEST

To explore the potential of using saving groups to increase access to maternal health services, the MANIFEST project worked in the selected groups in the intervention areas. Among other objectives, the MANIFEST project was implemented to increase birth preparedness and to increase access to household financing for maternal health and access to transport among pregnant women in the intervention area [20]. Strategies for increasing birth preparedness were encouraging people to join savings groups and working with saving groups on how they should save for maternal and newborn health [20]. The saving groups were also asked to make agreements with transporters so that the transporters could provide transport services to the women who were pregnant. Community development officers, who are government employees, provided technical support to the saving groups to strengthen their capacity to manage the saving groups.

### Sampling and data collection procedure

Data for this paper were obtained from a baseline survey that was done among 247 CBSGs. It included all the groups that were represented at the orientation training that was done for CBSG leaders at the start of the MANIFEST project. The community development officers at district and subcounty levels in each district purposively selected the CBSGs that were active at the time of the study. The study respondents were chairpersons and secretaries of savings groups, whom we believed had enough information on the operations of the CBSG. After consenting by signing or using thumbprint for those who could not sign, they were asked to complete self-administered questionnaires.

To gain more insight on the characteristics, challenges and management of savings groups, seven in-depth interviews were purposively conducted with chairpersons of the four registered and the three non-registered CBSGs. An interview guide was developed to guide the process of interviewing study participants and all interviews were audio-recorded.

### Study variables

To analyse the characteristics of the savings groups we collected data on the size of the groups, criteria for joining savings groups, frequency of meetings and savings, selection of the management committee, safety of savings funds and loan management (amount of money lent, pay back periods and interest rates). In addition, we collected information on the challenges faced by the CBSGs, as well as solutions.

### Data management and analysis

The quantitative data management and analysis was performed using SPSS version 20. The first author, with support from other team members, coded the open-ended questions after going through the questionnaire and having consensus on the codes for each question. Descriptive analysis was done to determine frequencies of different parameters.

For qualitative data, a few leaders were purposively selected given their roles and experience in managing CBSGs and written consent was sought before interviewing and recording their responses, although some could not sign and used a thumbprint. During data analysis, research assistants transcribed the audio recordings. To start the analysis, the first author read and re-read all seven transcripts. Next, identification of relevant sections, also referred to as meaning units in content analysis, was undertaken [21,22]. These sections described the organizational characteristics of the CBSGS. Thereafter, open coding was undertaken to the meaning units and a list of 80 open codes was generated. We grouped and re-grouped through iterative process, from which five main categories that describe the savings groups emerged. This process involved reviewing of the codes, subcategorizing and categorizing by MT, RMK and EEK to increase the credibility of the process. The main categories that are described in the results included: reasons and benefits for starting CBSGs; organizational profile of existing CBSGs; operations mechanisms of existing CBSGs; benefits of CBSGs to their members; challenges and solutions. Table 1 shows an example of how the qualitative analysis was carried out. In the results section, the qualitative results are used to illustrate and triangulate the quantitative findings. Therefore, both the quantitative and qualitative results are presented side by side in the results section.

**Table 1.** Example of the analysis process.

Text	Open codes	Subcategories	Category
Men are very difficult to trust, they are very clever, they will lie to you and misuse your money. So we don’t trust men and we cannot give them our money unless his wife guarantees that it will be paid. We have a challenge of poor management skills, we do not have anyone to train us. If government could support us we could be very happy. Nowadays we are also dealing with only people we know, so that we can be transparent with each other. We need trustworthy people and honest ones But the police and other district people can also help us when we have problems, especially if you are registered.	Loan defaulting, Dishonesty is a threat to groups, Fraud, Men are not trusted, Failing to complete a group constitution, High registration demands, Transparency attracts members, Using government structures to resolve conflicts Limited amounts of money to be borrowed to manage loan defaults, building trust, praising honesty	Dishonesty and Fraud Lack of capacity-building support Heavy registrationrequirements Building trust and honesty Using government structures	Challenges and solutions

### Ethical considerations

This study is part of the MANIFEST research protocol that was registered with Makerere University School of Public Health Research Ethics Committee (reference number HDREC 152) and the Uganda National Council for Science and Technology (reference number HS 1399). All participants provided written informed consent by signing consent forms or using a thumbprint for the few cases that could not write, and confidentiality and voluntary participation were assured.

## Results

The results of this paper are organized according to categories developed during the qualitative analysis. Under the categories, we first present the quantitative results and then follow with the qualitative results.

### Reasons and benefits for starting CBSGs

Table 3 describes the reasons for which CBSGs were started and the benefits of the CBSG to the members. The main reasons for the formation of CBSGs were to increase household income (84%), developing the community (18%) and saving for emergencies (8%). The most common benefits mentioned included earning dividends from savings (97%) and obtaining low-interest loans (66%). The other benefits included improving household welfare (22%), promoting social cohesion (29%) and meeting health needs (18%). The in-depth interviews with the CBSG leaders revealed that the need to develop social capital and self-improvements were at the centre of starting savings groups. Informants noted the need to belong, meeting each other’s needs and the desire to join hands in times of adversity as key to developing their social capital. To some of the informants, the social cohesion that came with starting savings groups was much more appreciated, compared to the accruing benefits. On the other hand, benefits such as social support, earning dividends and income generation were noted. The quotes below exemplify the reasons for setting up savings groups and the perceived benefits.

'The members get things to use, we get friendship … we help each other. When I lose someone or get a problem, they say that a colleague from Kibuku … lost a wife, or child, or father or mother, then the funeral rites or burial will be well attended. People will come in and help.' (CBSG leader 1)

'In our group, we deposit the money every Saturday during the meetings, and then lend it out same day, for example in case someone has a financial problem may be of school fees or he is sick, he can borrow and they bring back the money with a profit. For example, I joined this group after acquiring land around, but now I have been able to construct a house using my savings plus the soft loans.' (CBSG leader 3)

**Table 2.** Formation and management characteristics of CBSGs.

Variables	Number (%)
**Over all**	**247 (100)**
**Size of CBSG**	
15–30	141 (57%)
5–14	66 (27%)
>30	40 (16%)
Criteria for joining CBSGs*	
By registration (after paying)	236 (96%)
Community member	17 (7%)
Age consideration	15 (6%)
By buying shares	12 (5%)
**Frequency of depositing savings and group meetings**	
Weekly	22 (9%)
Bi-monthly	92 (37%)
Monthly	112 (45%)
Quarterly and above	21 (9%)
**How management committee is elected**	
Election/voting	232 (94%)
Nomination of members	15 (6%)
Safety of CBSG funds*	
Metallic box (safe box)	220 (89%)
All money is lent out	37 (15%)
Bank account/mobile money	24 (10%)
**Loan management**	
*Minimum amount of loan given to member†*	
$2–$5	212 (85%)
$6–$8	35 (14%)
*Loan payback period*	
Within 1–2 months	168 (68%)
Above 2 months	79 (32%)
*Interest rates for loans per month*	
6–10%	201 (81%)
< 6%	42 (17%)
> 11%	4 (2%)

### Organizational profile of existing CBSGs

Table 2 describes the organizational aspects of the existing CBSGs in the three districts studied. Over a quarter (27%) of the CBSGs had membership of 5–14 and slightly more than half (57%) of the CBSGs had between 15 and 30 members. Findings from the qualitative data indicated that most of the groups had an average size of 30 persons and women dominated the composition in terms of sex. It was noted that women were generally in leadership positions of the groups, mainly because they did not trust the men with the savings. However, in groups that had men, men also tended to occupy leadership positions. As a general observation, the informants noted that groups headed by men tended to fail because of mistrust and mismanagement of savings.

'We had a group of only men and three women, but that group died out in December last year; they had only like three months of existence. Men are not easy to work with, they can confuse you and steal your money; so people do not trust men.' (GBSG leader 3)

### Registration of savings groups

Registration of savings groups was noted as occurring at both district and subdistrict levels. The cost of registration varied according to level of registration in the three districts studied. At the subdistrict level, which was the first level of registration, it ranged between sh20,000 ($7) and sh50,000 ($17.8), while at district level the cost ranged between sh50,000 ($17.8) and sh100,000 ($35.7). The key informants noted that in addition to individual membership, local authorities required official registration of the CBSGs, but this was often resisted and undertaken reluctantly, because it was considered too costly. The registered CBSGs were legally bound by existing laws and were recognized by the subcounty and district leadership. In case of default from payment, they sought support from the legal structures in the district. On the other hand, the unregistered CBSGs members had no legal protection and relied on mutual understanding. The registered groups also benefit from the few grants provided by the central government to the local government. Some of the reasons that were given for failure to register included high registration fees and lack of constitutions, which are required at registration.

'The problem we have now is our constitution is not yet finalized because they want sh70,000 ($30) from us to complete it. Then after that the registration fees are also high, but anyway we shall try to register, because it also has its own benefits, like being trusted by members, getting help from police, in case of problems.' (CBSG leader 7)

### Operations mechanisms for existing CBSGs

Under operations of existing CBSGs, we consider the criteria for joining savings groups, frequency of depositing savings and meetings, selection of the management committee, means of ensuring funds safety and loan management.

#### Criteria for joining savings groups

Table 2 indicates that over 95% of the groups had registration as a means of joining the groups. Other means of joining included buying of shares, being 18 years and above and being a community member. Similarly, the key informants noted that membership fees were levied on members at registration. These fees were noted as important in meeting the operational costs of the groups. As in the quantitative findings, most groups accepted members who were above 18 years because they were considered old enough to be responsible. However, one of the leaders noted that this leads to exclusion of those below 18 from saving groups. In addition, community membership and one’s character were assessed before recruitment into a group, according to the informants. The quotes below exemplify the criteria for joining savings groups.

'Many willing members that are under 18 years are not allowed to join CBSGs for fear that they do not have any security or they are not yet sure of what they want to do with their money. This has been unfair to some of the young ambitious and hardworking youth in our communities.' (CBSG leader 5)

'If you come to me and express your interest to become a member, I take it to the members to debate on whether you are fit to be a member and whether, according to our assessments, you will not disturb us. Then we allow you. Usually you need to be a well-behaved member of our community. We don’t work with people we do not know.' (CBSG leader 7)

**Table 3.** Reasons and benefits for CBSGs.

Variables	Number (%)
**Overall**	**247 (100%)**
Reasons for joining or starting CBSGs*	
Improving household savings/income for, e.g., school fees	207 (84%)
Contributing to the development of area/community	45 (18%)
Addressing emergencies such as death and health	19 (8%)
Benefits provided by CBSGs to group members*	
Earning dividends from savings	240 (97%)
Obtain low-interest loans	162 (66%)
Promotes social cohesion during crises such as death	71 (29%)
Improves household welfare/improved standards of living	53 (22%)
Assists with meeting health needs	45 (18%)
Access to school fees	17 (7%)
Promotes teaching hygiene/sanitation	9 (4%)
Provides security for savings	4 (2%)

*Multiple answers.

#### Frequency of savings and meetings

Frequency of savings and meetings was reported as majorly monthly (45%) and bi-monthly (37%) (Table 2), while the others saved and met either weekly and rarely quarterly. The qualitative informants generally confirmed these findings and noted that the savings were also categorized into two accounts – the loaning account and welfare accounts. The loaning account was generally used for income-generating activities and borrowing from this account attracted interest, while the welfare account was meant to meet the social emergencies of members. Borrowing from the social welfare account did not attract any interest.

About selection of the management committee, 94% of the CBSGs indicated electing their management committees democratically (Table 2). The qualitative interviews revealed that aspects of trustworthiness and honesty were always considered when selecting members to leadership positions. These attributes were essential in securing the members’ savings, but also in building the credibility of the groups for purposes of growth and expansion. Every committee was usually composed of a chairperson, a vice chairperson, a treasurer and a secretary. The chairperson offered general oversight to the group, with the assistance of their vice chairperson, while the secretary ensured that the group records were kept. The treasurer was responsible for the accounting and safety of funds. The safety of the funds was nonetheless a collective responsibility, as shall be seen under the subcategory of ensuring funds safety.

'For us we select our leaders every year. But the leaders must be members for at least a year and … we must know you before you lead us: your character, the way you pay your loans and so on. Leaders are the ones who give us direction, so they have to be trusted people.' (CBSG leader 4)

Safety of funds was assured in several ways. According to the quantitative assessment, 15% of the CBSGs had lent out all their savings while some (89%) kept their money in a metallic box to ensure safety (Table 2). The informants also acknowledged that the pooled funds were mostly kept in metallic boxes that had three padlocks whose keys were kept by different members of the committee or group members at times. They also explained that when the money accumulated, several members rather than one person kept it. This was meant to secure the safety of the pooled funds and to increase trust in the organization of the savings groups. The quotes below highlight these issues.

'The treasurer keeps our metallic box, but … I have one key for that box, so it’s difficult for him to open the box without other members being around. Even the secretary keeps another key and we do not stay near each other.' (CBSG leader 7)

'… we keep it in a box and when the money accumulates because of returns from the loans advanced, we decide that each person keeps a portion of the money, otherwise the treasurer will be at risk of keeping very large sums of money.' (CBSG leader 1)

Lastly, in terms of loan management, we look at the minimum amount of money a member could borrow, the loan payback period and the interest rates. Minimum loan amounts ranged from $2 to $8. Most groups (81%) gave loans at an interest rate of 6%–10% per month and most of them wanted the loan to be paid back within one to two months (see Table 2). The informants noted that loans were loaned mainly to group members and rare exceptions were made to non-members. In such rare cases, the interest rates were usually higher and more strict terms applied; for example, identification of a guarantor or providing specific security. In addition, it was revealed that in groups that had both men and women, women tended to be more compliant to loan repayment schedules, while men tended to default. It was, therefore, taken as a caution to limit loans to men and to require women to serve as guarantors for their spouses. The quote below illustrated some of the loan management mechanisms in CBSGs.

'That is why we have instituted a maximum of six men in a group. Men are hard to manage, they default a lot, but a woman, even if she borrowed the money on behalf of her husband, she would pester him until he pays it back fully. That is why we have few men. It’s for our own good.' (CBSG leader 5)

### Challenges faced by existing CBSGs and solutions suggested

The most common challenges faced by the CBSGs included interpersonal relationships (34%), irregular attendance of group meetings (22%), lack of training in financial management (19%), registration challenges (15%) and failure to recover the loans (13%). Table 4 indicates the challenges faced by CBSGs. The in-depth interviews confirmed that the challenges were dominated by: issues of fraud, loan defaulting, high registration fees and requirements at the subcounty and district levels, a lack of regular training and capacity-building support to the savings groups. The quotes below illustrate some of the challenges.

'When you lend them money, at times they may default, and then you have to struggle to recover that money.' (CBSG leader 5)

'These men are very difficult; you know they like to try many different things. Therefore, you find that money can be lost and they can be arrogant when you ask them for the money.' (CBSG leader 6)

**Table 4.** Challenges faced by CBSGs.

Variables	Number (%)
**Overall**	**247 (100%)**
Interpersonal relationships	86 (35%)
Irregular attendance of group meetings	54 (22%)
Lack of training in management and administration	47 (19%)
Registration challenges (documentation and fees)	38 (15%)
Loss of group assets (e.g. deaths of animals)	34 (14%)
Failure to recover loans	32 (13%)
Lack of support	16 (7%)
Poor/small savings	16 (7%)
Membership dropping out	15 (6%)
Political interference with operations	4 (2%)
Lack of security	3 (1%)

Some codes had multiple responses.

Despite these challenges, the CBSG leaders suggested some solutions to deal with these shortcomings. Table 5 summarizes the solutions suggested by the group leaders. The most common solutions suggested were having good management (33.2%), timely payment of loans (28%), financial management training (22%), government support (17%) and engaging in income-generating activities (15%). The qualitative informants suggested three broad categories of solutions, which included using government structures to secure group funds and to improve skills development, as well as building trust and honesty among members. This was envisioned to build a strong social network among group members to increase transparency and responsibility. This was captured in the quotes below.

'We need to work with only close members of our community. Because it is these people we don’t know well who are spoiling our name. They come borrow money and just disappear and then come back after a long time.' (CBSG leader 2)

'The district should also help us. For us who are registered, the police can help at least in recovering our money. If people know that the government is behind us, they will fear to steal our money.' (CBSG leader 3)

**Table 5.** Group leaders suggested solutions for addressing CBSGs challenges.

Variables	Number (%)
**Overall**	**247 (100%)**
Good management	82 (33%)
Payment of loans on time	68 (28%)
Financial management training/sensitization/seminars	53 (22%)
Support from government/NGOs/well-wishers	42 (17%)
Engaging in income-generating activities	36 (15%)
Buying shares	27 (11%)
Exchange visits	24 (10%)
Saving groups registration	18 (7%)
Awarding best-performing groups	12 (5%)
Providing security/loans	12 (5%)
Increasing interest rates/member fees	10 (4%)

*Multiple responses.

## Discussion

In the discussion, we reflect the results in relation to existing literature. This will be organized according to the four main categories: reasons for starting CBSGs and their benefits, organizational profile of existing savings group, operations mechanisms of existing savings groups and the challenges faced by existing savings groups and suggested solutions.

### Reasons for starting CBSGs and their benefits

As can be seen from the findings, majority of the CBSGs were formed to fight rampant poverty in the communities through saving and starting income-generating activities. In other studies it has been observed that the groups promote savings and income-generating activities as a means of fighting household poverty [7,23]. Studies on CBSGs in developing countries have indicated that saving groups do not only improve immediate livelihoods, but also have a positive impact on children’s education, households’ health status and other individual and collective needs [7,12,24–27]. For emergencies such as death and illness, funds were loaned to members at no interest. One out of five CBSGs indicated meeting health needs as one key benefit to its members. This, therefore, presents an opportunity that could be exploited to increase savings meant to meet health needs, given that members already view CBSGs as a source of funds for dealing with emergencies. However, this comes with challenges of either delays or non-payment of loans given out to members due to emergency health needs or other home demands.

Community solidarity and cohesion were also reported as accruing from membership to CBSGS. This could be because different members get to know their village mates better and this creates a better understanding of their economic status. Community social networks have been reported as useful in providing safeguards to households against aftershocks, such as chronic sicknesses and death [23]. This attribute of the CBSGs identified in this study is an opportunity that could be harnessed to improve access to maternal and neonatal health services, as community members work together in groups to break financial barriers to accessing care in a timely manner and creates a better understanding of other members’ situations.

### Organizational profile of existing CBSGs

The majority had 5–30 members, had a management team that was selected democratically and met regularly. Groups of less than 30 are recommended because smaller groups are easier to manage and meetings also take a shorter time when the members are few [7]. However, about 16% of the saving groups had more than 30 group members. This could pose a challenge to management and could be related to the failures of such groups.

The democratic election processes used to select leaders allow the election of leaders who are trusted members of the community and who can ensure the safety of the members’ savings. This created a sense of security among the members, an attribute that is important for such groups, given the limited access to more advanced means to safeguard their savings. CBSGs are therefore encouraged to keep membership within known members, but also have high moral standards for its leaders to ensure survival and profitability.

### Operations mechanisms for existing CBSGs

The saving groups had various criteria for joining saving groups. One of the common ones included being above 18. Those below 18 were often excluded because they did not have the resources that would allow them to join the group, such as membership fees and security in case they required a loan. Secondly, they were considered immature because they are still children, according to the Uganda constitution, which stipulates 18 years as the age of majority. This leads to the exclusion of vulnerable groups such as teenage mothers, and yet these are some of the community members that could benefit from the services offered by saving groups, because they often have limited support [28,29]. Women groups could, therefore, be encouraged to include pregnant teenagers, even if they are younger than 18. Such teenagers and other vulnerable groups who are excluded for similar reasons could also be given subsidies so that they are able to afford the membership fees.

Most CBSGs kept their money in metallic safe boxes, while those without metallic boxes kept their money with the treasurer. A small percentage indicated saving using the mobile money technology. Keeping funds with treasurers or in metallic boxes may be risky when the amount of money is large [7]. The use of mobile money technology is safer [7]; however, a reflection on its applicability in rural communities is essential. Rural communities often have limited mobile phone network and infrastructure coverage. In addition, the mobile money technology could be challenged by the scarcity of electricity needed to charge mobile phones and the relatively low levels of literacy in rural communities. Nevertheless, access to mobile phones is steadily increasing in developing countries, as more suppliers come on board. Secondly, mobile solar technology which can be used for charging is now available, although its use is still limited in rural areas. Although saving groups have been known to keep very basic records, as groups grow and their transactions increase, the need for some financial management skills increases. As noted earlier, many groups reported that many of their leaders had not been trained in financial management and other income-generating activities. Governments should put in place mechanisms for providing technical support to these groups.

### Challenges faced by existing CBSGs and solutions suggested

Despite the important contributions of the CBSGs, several challenges were noted that hinder their progress. These challenges include failure to recover the loans from members mainly due to poverty or poor agricultural yields, lack of training in financial management and irregular attendance of group meetings, which at times led to unfair decisions by the few members present. In addition, there were registration challenges due to the small savings, fraud, theft and high illiteracy levels among members, which rendered them weak when it came to group organization and management.

High costs of registration at subcounty and district level were also noted to be a problem. Yet the savings groups are mandated to be registered at the subcounty and the district development office if they are to gain from government community development interventions. In addition, registered savings groups are recognized and they are helped in case some members default. The costs for registration should be reduced so that it is easier for the saving group members to register, which will allow them to become legal entities and it will enable them to seek redress when they experience difficulties.

Registration should also entitle them to more technical support from community development officers. Furthermore, an increase in the number of grants given to such community-based organizations is also recommended, because that can serve as an incentive for improved performance and can provide capital needed for investment for the groups. This would allow the groups to generate more income and hence contribute more significantly to increasing household incomes.

### Study strengths and limitations

Our findings have some limitations. First, the data reviewed were collected from CBSGs that were selected purposively by the district leaders. This may have introduced some selection bias. Furthermore, information was collected from CBSGs in rural communities of Eastern Uganda whose reasons for saving are based on these community cultures and characteristics. Therefore, the results of this study can only be generalized to areas with a similar setting. Additionally, this study only interviewed the leaders of the CBSGs and therefore the benefits, management capacity and challenges may not reflect the perceptions of the group members, thus suggesting a need for a comprehensive study that involves the group members. A few of these leaders could not write or sign and therefore used the thumbprint to signify consent. Lastly, our results may have been affected by the clustering effect of villages within a district. We did not consider the district as a study domain, so we were not able to compare the savings groups across the three districts and so we may have missed some contextual differences between the districts. However, as the districts belong to the same region, the contextual differences are most likely minimal.

## Conclusions

There are already many saving groups in rural communities. These CBSGs could, therefore, be harnessed for the benefit of maternal and newborn health as a source of immediate financial support, which is crucial, especially during emergencies. More awareness about the benefits of belonging to these CBSGs could encourage more enrolment. Nonetheless, these CBSGs need to be supported by relevant government departments and NGOs to enable them tackle existing challenges.

## Paper context

Health facility utilization in eastern Uganda, where this study was conducted, was below the national estimates (52% for health facility delivery and 36% postnatal care) [30]. In these communities, lack of money for transportation and payment for other health-related costs is one of the reasons that lead to underutilization of maternal and newborn services [4,31]. This article explores the possibility of using CBSGs in increasing birth preparedness in rural communities of Uganda.
